# Copy number variations in *RNF216* and postsynaptic membrane–associated genes are associated with bipolar disorder: a case‐control study in the Japanese population

**DOI:** 10.1111/pcn.13752

**Published:** 2024-10-15

**Authors:** Masahiro Nakatochi, Itaru Kushima, Branko Aleksic, Hiroki Kimura, Hidekazu Kato, Toshiya Inada, Youta Torii, Nagahide Takahashi, Maeri Yamamoto, Kunihiro Iwamoto, Yoshihiro Nawa, Shuji Iritani, Nakao Iwata, Takeo Saito, Kohei Ninomiya, Tomo Okochi, Ryota Hashimoto, Hidenaga Yamamori, Yuka Yasuda, Michiko Fujimoto, Kenichiro Miura, Kazutaka Ohi, Toshiki Shioiri, Kiyoyuki Kitaichi, Masanari Itokawa, Makoto Arai, Mitsuhiro Miyashita, Kazuya Toriumi, Tsutomu Takahashi, Michio Suzuki, Takahiro A. Kato, Shigenobu Kanba, Hideki Horikawa, Kiyoto Kasai, Tempei Ikegame, Seiichiro Jinde, Tadafumi Kato, Chihiro Kakiuchi, Bun Yamagata, Shintaro Nio, Yasuto Kunii, Hirooki Yabe, Yasunobu Okamura, Shu Tadaka, Ueno Fumihiko, Taku Obara, Yasuyuki Yamamoto, Yuko Arioka, Daisuke Mori, Masashi Ikeda, Norio Ozaki

**Affiliations:** ^1^ Public Health Informatics Unit, Department of Integrated Health Science Nagoya University Graduate School of Medicine Nagoya Japan; ^2^ Department of Psychiatry Nagoya University Graduate School of Medicine Nagoya Japan; ^3^ Medical Genomics Center Nagoya University Hospital Nagoya Japan; ^4^ Department of Child and Adolescent Psychiatry Nagoya University Graduate School of Medicine Nagoya Japan; ^5^ Okehazama Hospital Brain Research Institute Toyoake Japan; ^6^ Department of Psychiatry Fujita Health University School of Medicine Toyoake Japan; ^7^ Department of Pathology of Mental Diseases, National Institute of Mental Health National Center of Neurology and Psychiatry Kodaira Japan; ^8^ Department of Psychiatry Osaka University Graduate School of Medicine Suita Japan; ^9^ Japan Community Health care Organization Osaka Hospital Fukushima Japan; ^10^ Life Grow Brilliant Mental Clinic Medical Corporation Foster Osaka Japan; ^11^ Department of Psychiatry Gifu University Graduate School of Medicine Gifu Japan; ^12^ Department of General Internal Medicine Kanazawa Medical University Uchinada Japan; ^13^ Laboratory of Pharmaceutics, Department of Biomedical Pharmaceutics Gifu Pharmaceutical University Gifu Japan; ^14^ Vice Director General Tokyo Metropolitan Institute of Medical Science Tokyo Japan; ^15^ Schizophrenia Research Project, Department of Psychiatry and Behavioral Sciences Tokyo Metropolitan Institute of Medical Science Tokyo Japan; ^16^ Department of Psychiatry Tokyo Metropolitan Matsuzawa Hospital Tokyo Japan; ^17^ Department of Psychiatry Takatsuki Clinic Akishima Japan; ^18^ Department of Neuropsychiatry University of Toyama Graduate School of Medicine and Pharmaceutical Sciences Toyama Japan; ^19^ Research Center for Idling Brain Science University of Toyama Toyama Japan; ^20^ Department of Neuropsychiatry, Graduate School of Medical Sciences Kyushu University Fukuoka Japan; ^21^ Japan Depression Center Tokyo Japan; ^22^ Horikawa Hospital Kurume Japan; ^23^ Department of Neuropsychiatry, Graduate School of Medicine The University of Tokyo Tokyo Japan; ^24^ The International Research Center for Neurointelligence at The University of Tokyo Institutes for Advanced Study Tokyo Japan; ^25^ Department of Psychiatry and Behavioral Science Juntendo University Graduate School of Medicine Tokyo Japan; ^26^ Department of Neuropsychiatry Keio University School of Medicine Tokyo Japan; ^27^ Department of Psychiatry Saiseikai Central Hospital Tokyo Japan; ^28^ Department of Disaster Psychiatry, International Research Institute of Disaster Science Tohoku University Sendai Japan; ^29^ Department of Mind & Brain Medicine Fukushima Medical University Fukushima Japan; ^30^ Advanced Research Center for Innovations in Next‐Generation Medicine Tohoku University Sendai Japan; ^31^ Tohoku Medical Megabank Organization Tohoku University Sendai Japan; ^32^ Center for Advanced Medicine and Clinical Research Nagoya University Hospital Nagoya Japan; ^33^ Brain and Mind Research Center Nagoya University Nagoya Japan; ^34^ Pathophysiology of Mental Disorders Nagoya University Graduate School of Medicine Nagoya Japan

**Keywords:** array comparative genome hybridization, bipolar disorder, pathogenic copy number variation, *RNF216*, synaptic gene

## Abstract

**Aim:**

Bipolar disorder (BD) is a common psychiatric disorder characterized by alterations between manic/hypomanic and depressive states. Rare pathogenic copy number variations (CNVs) that overlap with exons of synaptic genes have been associated with BD. However, no study has comprehensively explored CNVs in synaptic genes associated with BD. Here, we evaluated the relationship between BD and rare CNVs that overlap with synaptic genes, not limited to exons, in the Japanese population.

**Methods:**

Using array comparative genome hybridization, we detected CNVs in 1839 patients with BD and 2760 controls. We used the Synaptic Gene Ontology database to identify rare CNVs that overlap with synaptic genes. Using gene‐based analysis, we compared their frequencies between the BD and control groups. We also searched for synaptic gene sets related to BD. The significance level was set to a false discovery rate of 10%.

**Results:**

The *RNF216* gene was significantly associated with BD (odds ratio, 4.51 [95% confidence interval, 1.66–14.89], false discovery rate < 10%). The BD‐associated CNV that corresponded with *RNF216* also partially overlapped with the minimal critical region of the 7p22.1 microduplication syndrome. The integral component of the postsynaptic membrane (Gene Ontology:0099055) was significantly associated with BD. The CNV overlapping with the intron region of *GRM5* in this gene set showed a nominal significant association between cases and controls (*P* < 0.05).

**Conclusion:**

We provide evidence that CNVs in *RNF216* and postsynaptic membrane–related genes confer a risk of BD, contributing to a better understanding of the pathogenesis of BD.

Bipolar disorder (BD) is a common psychiatric disorder characterized by mood swings between manic/hypomanic and depressive states.^1^ The mood episodes can last for days to months and impact an individual's daily functioning, relationships, and quality of life considerably. Despite the high prevalence and substantial burden of BD, its precise cause remains unclear. However, evidence suggests that genetic factors play a crucial role in the development and progression of BD. Its lifetime prevalence is >0.4%,^2^ and twin studies estimate the heritability of BD to be >60%.^3^, ^4^, ^5^ Despite the high heritability, current diagnostic and treatment approaches for BD are limited by the lack of reliable biomarkers, highlighting the need for a better understanding of the underlying genetic mechanisms.

Single‐nucleotide polymorphism (SNP)–based heritability on the liability scale is estimated to be 14.8%^6^ and 18.6%^7^ in the Japanese and European populations, respectively. Genome‐wide association studies (GWASs) have identified more than 50 loci associated with BD.^6^, ^7^, ^8^, ^9^, ^10^, ^11^ One of the largest GWASs for BD reported that BD risk alleles were enriched in genes in synaptic signaling pathways and brain‐expressed genes, particularly those with high specificity of expression in neurons of the prefrontal cortex and hippocampus. However, a polygenic risk score for BD constructed based on a GWAS in a European population^7^ explained only 4.57% of the phenotypic variance in BD on the liability scale. Therefore, there is a considerable difference in heritability between SNP‐based studies and other studies. This may be because genetic variants other than SNPs, such as rare variants, which contribute strongly to BD, have not been identified.

Copy number variations (CNVs), structural genomic variations typically larger than 50 bp that occur as deletions, and duplications and insertions have been implicated in the risk of psychiatric disorders, including schizophrenia (SCZ), autism spectrum disorder (ASD), and others.^12^ Relatively large, rare CNVs (>500 kb and present in <1% of the population) are particularly associated with an increased risk of such disorders.^12^, ^13^, ^14^, ^15^, ^16^, ^17^, ^18^, ^19^, ^20^ Several studies have successfully identified CNVs that contribute to BD.^21^, ^22^, ^23^, ^24^, ^25^, ^26^, ^27^, ^28^ For example, a study analyzing CNVs in the Japanese population reported that CNVs at *DLG2*, *PCDH15*, and *ASTN2* are significantly associated with BD.^13^ Furthermore, gene‐set analysis in that study revealed enrichment of CNVs in genes related to chromatin biology, suggesting a potential role of chromatin regulation in BD pathogenesis. Although both ASD and SCZ were associated with synapse‐related pathways in the study, BD did not show a significant association.^13^ However, *DLG2* and *PCDH15*, identified to be associated with BD, are synaptic genes, suggesting that synaptic dysfunction may also be involved in BD pathogenesis. No previous association between BD and CNV has been evaluated for each gene involved in synapses.

Previous studies have focused on CNVs that overlap only with exon regions; however, CNVs in noncoding regions, including introns, are also associated with neurodevelopmental disorders.^13^, ^29^ It has been reported that intronic CNVs contribute to gene expression.^30^ The Synaptic Gene Ontology (SynGO) has been used frequently in CNV studies as a database for defining synapse‐related gene sets. SynGO gene sets are evidence‐based expert‐curated sets in synaptic biology.^31^ The latest version of SynGO, version 1.2, has recently been released. In the present study, we evaluated the association between CNVs in synaptic genes registered in SynGO version 1.2, regardless of exonic or intronic regions, and BD in Japanese individuals. The findings could enhance our understanding of BD pathogenesis.

## Methods

### Participants

The present study was a case‐control investigation performed based on a CNV data set obtained using array comparative genomic hybridization (aCGH). We recruited 4704 Japanese individuals, including 1857 patients with BD (42.2% with BD type I, 53.9% with BD type II, and 3.9% with an unknown subtype) and 2847 psychiatrically healthy controls (Supplementary Table S1). Among them, 1843 patients with BD and 1876 controls were included in our previous study,^13^ and samples were collected in the same manner as in that previous study.^13^ The CNVs were measured in individuals using an Agilent array, using aCGH, as described below. In addition, to assess whether the frequency of healthy individuals with each of the BD‐associated CNVs identified in this study was consistent among different aCGHs (Agilent and NimblGen arrays), we included an additional 837 independent validation samples that were used in our previous study.^13^ The CNVs of the validation samples were measured using a NimblGen aCGH.

The patients were diagnosed according to *DSM‐5* criteria for BD. Controls were selected from the general population and had no history of mental disorders, based on their responses to questionnaires or self‐report. The characteristics of the patients are described in our previous study.^13^ The present study was approved by the ethics committee of Nagoya University (No. 2010–1033) and its participating institutes. The study conformed to the provisions of the Declaration of Helsinki. Written informed consent was obtained from all participants.

### Array comparative genome hybridization

The CNV data set was analyzed using two types of aCGHs. First, the Agilent SurePrint G3 Human CGH 400K array (Agilent Technologies) was used for BD cases and controls. The NimbleGen 720k Whole‐Genome Tiling array (Roche NimbleGen) was used to validate the frequency of each BD‐associated CNV. A procedure similar to that in our previous study^13^ was used to measure the aCGH CNV calls made with Nexus Copy Number 9.0 (BioDiscovery) using the fast adaptive state segmentation technique 2 algorithm. The following log_2_ ratio thresholds were set to detect CNVs in the Agilent arrays: 10–500 kb: −0.6 (deletion) and 0.4 (duplication), >500 kb: −0.4 (deletion) and 0.3 (duplication). The significance threshold to adjust the sensitivity of the segmentation algorithm was set at 1 × 10^−6^, and at least three contiguous probes were required for CNV calls. A noise‐reduction algorithm for the aCGH data was used for systematic correction of artifacts caused by GC content and fragment length.^32^ We defined rare CNVs as CNVs in which the carrier frequency in the population was <1%. Quality control of aCGH and annotation of detected rare CNVs are described in the Supplementary Methods. Because both types of aCGHs used in this study were designed using hg18 as the reference genome, CNVs were detected using the hg18 genomic coordinate system. When displaying CNVs using Genome Browser, the coordinates of the detected CNVs were determined by converting the reference genome to hg38 using liftOver (https://genome-store.ucsc.edu/).

### Gene‐based analysis

To identify synaptic genes associated with BD, gene‐based analysis using the rare CNV data set was performed. For each synaptic gene registered in SynGO version 1.2,^31^ the frequency of CNVs overlapping with each synaptic gene was compared between patients with BD and healthy controls. Firth bias‐reduced logistic regression was used for comparison.^33^ The dependent variable was the case versus control status, and the independent variables included the presence or absence of overlapping CNVs for each synaptic gene with sex as a covariate in the model. To reduce the number of false positives, genes registered in SynGO with overlapping CNVs in at least 10 subjects were included in the analysis. As a result, 37 genes were included in the analysis. *P*‐values were corrected for a false discovery rate based on the Benjamini and Hochberg (BH) method^34^ to correct for multiple testing. A gene was considered significant if its Q‐value was <0.10. We also performed a comprehensive gene‐based analysis including genes not registered in SynGO to comprehensively search for BD‐associated genes. To reduce the number of false positives, genes with overlapping CNVs in at least 10 subjects were included in the analysis. In total, 686 genes were included in the analysis. Multiple correction was performed using the BH method.

Gene‐based analysis adjusted for pathogenic CNVs was performed. The details are provided in the Supplementary Methods.

### Gene‐set analysis

To identify the synaptic biological pathways underlying BD pathogenesis, we tested the enrichment of rare genic CNVs in synaptic gene sets relative to that of all rare gene CNVs. Details of the analysis are provided in the Supplementary Methods.

### Detection of CNVs overlapping with RNF216 from the WES data set of BD trios

To confirm the frequency of CNVs overlapping with *RNF216* in Japanese patients with BD, a whole exome sequencing (WES) data set from Japanese BD trios was used.^35^, ^36^ Details of the analysis are provided in the Supplementary Methods.

### Statistical analyses

The distribution of age in each group is summarized as the median (interquartile range). The distribution of sex in each group was summarized as the percentage of men. All statistical analyses were performed using R statistical software version 3.6 (https://www.r-project.org/).

## Results

### Detection of CNVs

We performed Agilent aCGH for 1857 patients with BD and 2847 controls. After QC, CNV data were obtained from 1839 patients with BD and 2760 controls (Supplementary Table S1). A total of 9781 rare CNVs were detected using Agilent aCGH. We detected 2.18 rare CNVs per BD case and 2.09 per control. Of the 9781 CNVs detected, 1623 rare CNVs were intronic CNVs.

### Synaptic gene‐based analysis results

**Fig. 1:**
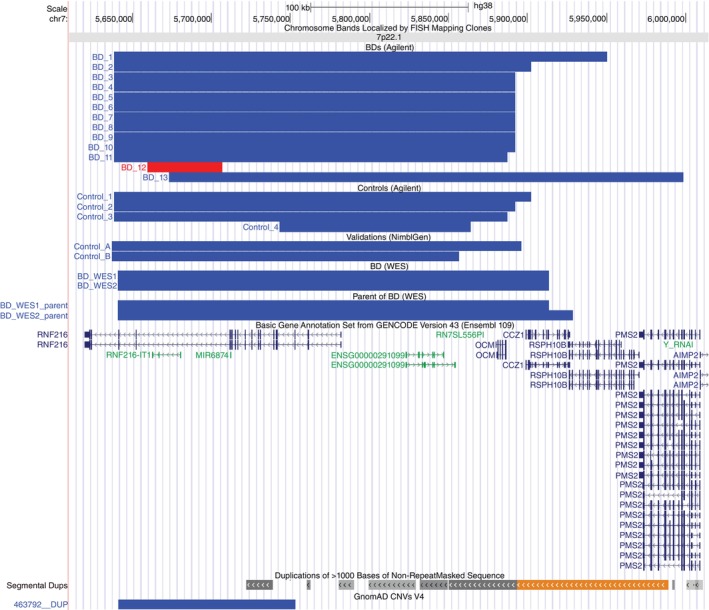
Copy number variations (CNVs) detected in *RNF216* in patients with bipolar disorder (BD) and controls. UCSC Genome Browser view for the region chr7:5,610,000–6,015,000. Genomic coordinates are based on hg38. The upper track shows the genomic locations of the detected duplications and deletions in the following order: patients with BD and healthy controls were evaluated using Agilent aCGH, validation samples were evaluated using NimblGen aCGH, and patients with BD and their parents were evaluated using whole exome sequencing (WES). Duplicates are indicated by blue bars and deletions by red bars. Sample numbers do not correspond to the sample numbers in Supplementary Fig. S1. The second track from the bottom shows annotations for SDs. The bottom track shows the gene annotations in Gencode V43 and 463792__DUP of gnomAD CNVs V4. Other variants registered in gnomAD are not shown.

For the 37 genes registered in SynGO, we compared the frequencies of carriers with CNVs that overlap with each of the synaptic genes between patients with BD and healthy controls, based on the Agilent aCGH data set. CNVs overlapping with *RNF216* were significantly associated with BD (Table 1). In addition, CNVs overlapping with *GRM5* had a nominally significant association with BD. CNVs in *RNF216* were detected in 13 patients (0.71%) and four control participants (0.14%). Of 17 CNVs overlapping with *RNF216*, 16 were duplications, and only one CNV from one patient with BD was a deletion (Fig. 1). CNVs in *GRM5* were detected in 25 patients (1.36%) and 19 control participants (0.69%). All 44 CNVs overlapping with *GRM5* were deletions located in the intron region and identified at nearly identical coordinates (Supplementary Fig. S1).

**Table 1:** Significant or nominally significant synaptic genes associated with BD

Ensembl gene ID	Gene symbol	Group	Platform	Frequency of CNV carriers (%)	OR (95% CI)^†^	*P*‐value^†^	Q‐value^†^
ENSG00000011275	*RNF216*	Case	Agilent	0.71	4.51 (1.66–14.89)	0.003	0.097
		Control	Agilent	0.14			
		Validation	NimblGen	0.24			
ENSG00000168959	*GRM5*	Case	Agilent	1.36	1.97 (1.09–3.61)	0.024	0.872
		Control	Agilent	0.69			
		Validation	NimblGen	0.73			

^†^ Odds ratios (ORs) and *P‐* and Q‐values were calculated based on the Agilent array comparative genome hybridization data set.

Abbreviations: BD, bipolar disorder; CI, confidence interval; CNV, copy number variation; OR, odds ratio.

We also obtained NimblGen aCGH data from 823 healthy participants after QC (Supplementary Table S1). We observed two carriers of CNVs overlapping with *RNF216* and six carriers of CNVs overlapping with *GRM5* and confirmed that the frequencies of the carriers were 0.24 and 0.73%, respectively (Table 1). These frequencies were similar to those of Agilent aCGH controls.

We evaluated whether the two identified genes were associated with BD independently of known pathogenic CNVs. The results of the gene‐based analysis after adding the presence or absence of pathogenic CNV as a covariate are shown in Supplementary Table S2. The results showed that both *RNF216* and *GRM5* remained significantly and nominally significantly associated with BD, respectively.

### Comprehensive gene‐based analysis results

No significant (Q<0.1) genes were identified from our comprehensive analysis (including genes not registered in SynGO), but 48 nominally significant genes were identified (Supplementary Table S3). The top four genes with the lowest *P*‐values were *RNF216‐IT1*, *OCM*, *RN7SL556P*, and *MIR6874*, all of which overlapped with CNVs containing *RNF216* (Fig. 1). *RNF216* had the eighth lowest *P*‐value.

### Clinical characteristics of patients with BD with RNF216 duplications

Among the 13 patients with BD who carried *RNF216* CNVs, clinical information was available for nine individuals with *RNF216* duplications (Table 2). Four patients were diagnosed with bipolar I disorder and five were diagnosed with bipolar II disorder. The age of onset varied from 19 to 56 years. Rapid cycling was observed in one patient, while psychotic features were present in five patients (55.6%). Three patients had a history of suicide attempts. Treatment included lithium, lamotrigine, atypical antipsychotics, and other mood stabilizers, with most patients showing a favorable response. Comorbid physical illnesses were reported in several patients, including diabetes, obesity, and hypertension.

**Table 2:** Clinical characteristics of patients with BD with *RNF216* duplications

Sample ID	BD_1	BD_4	BD_5	BD_6	BD_7	BD_8	BD_9	BD_10	BD_11
CNVs	*RNF216* duplication	*RNF216* duplication	*RNF216* duplication	*RNF216* duplication	*RNF216* duplication	*RNF216* duplication	*RNF216* duplication	*RNF216* duplication	*RNF216* duplication
Diagnosis	BD‐I	BD‐I	BD‐I	BD‐II	BD‐I	BD‐II	BD‐II	BD‐II	BD‐II
Age/sex	69/M	56/F	63/F	28/M	30/M	57/F	43/M	39/F	61/F
Family history	BD	Epileptic psychosis	SCZ	−	NA	NA	MDD	ASD, NDD	−
History of development	Normal	Normal	Normal	Normal	Normal	NA	NA	School refusal	NA
Age of onset	45	24	19	26	29	NA	25	27	56
Rapid cycling	−	−	+	−	−	−	NA	−	−
Psychotic features	+	+	−	−	+	−	−	+	+
Suicide attempts	−	−	+	−	−	−	−	+	+
Other clinical features	−	Panic attacks	Catatonia	−	−	Generalized anxiety disorder	−		Mild cognitive impairment
No. of hospitalization	10	3	>40	0	2	NA	0	Many	3
Treatment	Aripiprazole	Lithium	Lamotrigine, Aripiprazole	Sertraline, aripiprazole	Lithium, olanzapine	Mirtazapine, clonazepam	Duloxetine, olanzapine	Quetiapine, valproate, lamotrigine	NA
Treatment response	NA	Favorable	Lithium: Unfavorable; Lamotrigine: Favorable	Favorable	Favorable	Favorable	Favorable	Unfavorable	NA
Comorbidity of physical illnesses	−	Diabetes, obstructive sleep apnea syndrome	Diabetes, deep vein thrombosis	Fatty liver	Chronic sinusitis, urticaria	Hypertension, endocrine disorders	−	Obesity, iron deficiency anemia	Asthma

*Note*: This table summarizes the clinical characteristics of nine patients with bipolar disorder (BD) with *RNF216* duplications, including four patients with BD type I (BD‐I) and five patients with BD type II (BD‐II). Sample numbers correspond to the sample numbers in Fig. 1. Treatment response was based on the overall clinical assessment by the attending psychiatrist, considering the patient's symptomatic improvement and functional recovery.

Abbreviations: ASD, autism spectrum disorder; CNV, copy number variation; F, female; M, male; MDD, major depressive disorder; NA, not available; NDD, neurodevelopmental disorder; SCZ, schizophrenia.

### CNVs overlapping with RNF216 based on WES data set from Japanese BD trios

Using the previously reported WES data set of Japanese BD trios (N = 145 trios),^35^, ^36^ we attempted to detect CNVs that overlap with *RNF216*. We detected *RNF216* CNVs in a region similar to those of the CNVs identified in the Agilent aCGH from two of the 143 patients with BD (1.4%; Fig. 1). Similar CNVs were also detected in two of the 287 parents of patients with BD. This means that one of the parents of the patient with BD who had the *RNF216* CNV had the *RNF216* CNV as well. Neither of the two patients with *RNF216* CNV detected in the parent generation were patients with BD, but one (BD_WES1_parent in Fig. 1) had been diagnosed with a major depressive disorder.

### Comparison of carrier frequencies of CNVs overlapping with RNF216 across different or similar ancestry

Recently, the gnomAD project released rare (<1% overall carrier frequency) autosomal coding CNVs from WES in 464,297 individuals as part of gnomAD version 4 (https://gnomad.broadinstitute.org/). Based on the database, the CNV with accession ID 463792 _ DUP was confirmed. This CNV is located on chr7:5641057–5,753,077 (hg38) and part of the *RNF216* CNV that we identified (Fig. 1). The frequency of samples with this CNV was 0.156% in non‐neuro samples of the East Asian population, compared with a much lower percentage (<0.02%) in other populations (Supplementary Table S4).

Recently, the Tohoku Medical Megabank Organization (ToMMo) released frequencies of CNVs from whole‐genome sequencing (WGS) data in 48,874 Japanese individuals (https://jmorp.megabank.tohoku.ac.jp/). The number of CNV carriers per 1000 bases and their frequencies in the region shown in Fig. 1 are listed in Supplementary Table S5. As shown in Table 1, the frequency of CNVs overlapping with *RNF216* in our case and control groups measured by aCGH was 0.71% and 0.14%, respectively. Conversely, duplications or deletions overlapping with *RNF216* were observed in the ToMMo samples at a frequency of approximately 0.53% in the region spanning 5,636,501 to 5,897,500 bp on chromosome 7. Duplications and deletions were observed more frequently from 5,898,501 to 5,980,500 bp.

### Comparison of carrier frequencies of CNV overlapping with the intron region of GRM5 across different ancestries

Recently, the gnomAD project released genome‐wide SV from the genome sequencing of 63,046 individuals as part of gnomAD V4 (https://gnomad.broadinstitute.org/). According to the database, an SV (deletion) with accession ID DEL_CHR11_8173BAA3 was confirmed. This CNV is located at chr11:88745942–88,785,660 (hg38), which is almost identical to the position of the *GRM5* CNV we identified (Supplementary Fig. S1). The allele frequency for CNV was 0.123% in non‐neural samples from the East Asian population, and the carrier frequency for the CNVs was 0.247% (Supplementary Table S6). This CNV was not observed in the non–East Asian populations.

### Gene‐set analysis

We performed gene‐set analysis of 33 synaptic gene sets using SynGO version 1.2. As shown in Table 3, the integral component of the postsynaptic membrane (GO:0099055) showed a significant association with BD (odds ratio [OR], 1.70 [95% confidence interval (CI), 1.16–2.49], *P*= 0.003; Q= 0.089), and the postsynaptic membrane (GO:0045211) showed a nominally significant association with BD (OR, 1.40 [95% CI, 1.01–1.93], *P*= 0.021; Q = 0.672). The GO:0045211 is a parent term to GO:0099055. These GO terms included *GRM5* but not *RNF216*.

**Table 3:** Significant or nominally significant SynGO terms associated with BD

SynGO term	GO domain	Number of genes	Number of genes hit by CNVs	Group	Frequency of genes hit by CNVs per sample (%)	OR (95% CI)^†^	*P*‐value^†^	Q‐value^†^
Integral component of postsynaptic membrane	CC	120	28	Case	3.53	1.70 (1.16–2.49)	0.003	0.089
GO:0099055				Control	1.70			
Postsynaptic membrane	CC	164	40	Case	4.30	1.40 (1.01–1.93)	0.021	0.672
GO:0045211				Control	2.61			

*Note*: Odds ratios (ORs) represent the increased risk of bipolar disorder (BD) per gene hit by copy number variations (CNVs) for the SynGO term.

Abbreviations: CI, confidence interval; GO: gene ontology.

^†^ ORs and *P‐* and Q‐values were calculated based on the Agilent array comparative genomic hybridization data set.

## Discussion

It has been reported that SCZ and ASD are associated with CNVs that overlap with synaptic genes, and similar associations have been reported in BD.^13^ To identify additional BD‐associated synaptic genes, we evaluated their relationship with synapse‐related genes registered in the latest version of SynGO in 1839 Japanese patients with BD and 2760 controls. Gene‐based analysis identified CNV regions in *RNF216* as those significantly associated with BD. In addition, a CNV in the intron of *GRM5* was nominally significantly associated with BD. We also performed a comprehensive gene‐based analysis, and *RNF216‐IT1*, *OCM*, *RN7SL556P*, and *MIR6874* were found to have the lowest *P*‐values. These genes contained CNVs that overlapped with *RNF216*. This indicated that the region containing RNF216 was most strongly associated with BD from a genome‐wide perspective. However, our comprehensive gene‐based analysis did not identify any significant genes. This means that the sample size was insufficient for a genome‐wide approach. It would be reasonable to adopt synaptic gene‐based analysis as the primary analysis method to increase the detection power.

Furthermore, the gene‐set analysis revealed that a SynGO term (integral component of the postsynaptic membrane, GO:0099055) was significantly associated with BD. *GRM5* is included in this synaptic gene set. Our previous studies focusing on CNVs that overlapped with specified exonic regions failed to identify these BD‐associated CNVs and gene sets. The results suggested that noncoding regions such as introns are also involved in BD pathogenesis.^13^

Large‐scale studies on CNVs in BD have been conducted primarily in Western populations,^21^, ^22^, ^23^, ^24^, ^25^, ^26^, ^27^, ^28^ and CNVs in *RNF216* and *GRM5*, which were found in this study, were not identified. According to the GnomAD V4 database, the frequency of carriers of duplicate CNVs in *RNF216* is higher in East Asian populations than in European populations. In addition, deletion of CNVs in the *GRM5* intron was observed only in East Asians. This clear difference in frequency suggests that this CNV is specific to East Asian or Japanese populations and was not identified as being associated with BD in previous European studies. The CNVs identified in this study were rare, with <1% of the patients being carriers. In contrast, a certain number of CNVs were detected in the WES data of East Asians with GnomAD in the same region as this CNV. The CNVs, including in *RNF216*, were also detected in WES from Japanese BD trios for a parent and child in two families. Based on these observations, it is expected that these CNVs are not *de novo* mutations but a genomic variation inherited from the parent. Thus, if SNPs in linkage disequilibrium with these CNVs exist, we can expect them to be reported in future GWASs of East Asians. The largest GWAS in the East Asian population was reported by Ikeda et al. and Li et al.,^6^, ^9^ but these regions have not been reported as BD‐associated loci. It is conceivable that the sample size was not sufficiently large to detect these loci. Future GWASs of BD with larger East Asian sample populations might detect these identified regions. Recently, a GWAS of BD and major depressive disorder reported that rs4753209 is located in the intron region of *GRM5*.^37^ The locations of this primary SNP and the identified CNV were approximately 250 kb apart. This GWAS was conducted primarily in a Western population. According to GnomAD V4, the identified CNVs appeared to be East Asian–specific and therefore independent of the signals identified in this GWAS.

As shown in Fig. 1, CNVs overlapping with *RNF216* reported in GnomAD V4 are shorter than the CNVs we detected. This may be attributable to the presence of segmental duplications (SDs) in the region on the right side of Fig. 1; GnomAD V4 detects CNVs in the exon regions captured by the WES kit but may not have made CNV calls in the region overlapping with the SDs. Therefore, it is likely that the CNVs detected by GnomAD V4 are shorter than the CNVs that we detected.

In the Japanese WGS‐based CNV database published by ToMMo, a duplication and deletion frequency of approximately 0.53% was observed in the 5,636,501 to 5,897,500 bp region of chr7, which is lower than the frequency in our case samples, but higher than that in the control samples. This may be because of individuals from ToMMo with a history of psychiatric disorders being included. In addition, the difference could be attributable to the difference in measurement methods between WGS and aCGH. CNVs were more frequently detected in the 5,898,500 to 5,980,500 bp region of the same chromosome. The latter region particularly overlapped with SDs (Fig. 1), and it is possible that CNVs are not correctly detected.

Duplication of 7p22.1, which involves *RNF216*, is known to cause 7p22.1 microduplication syndrome,^38^, ^39^, ^40^ which is mainly characterized by developmental and speech delays, craniofacial dysmorphisms such as macrocephaly, hypertelorism, ear anomalies, and skeletal abnormalities.^41^ The minimal critical region of duplication associated with this syndrome is chr7:5497216–5,760,088 in hg38.^40^ This region and the identified BD‐associated CNVs partially overlapped in the region containing *RNF216*. The clinical manifestations of 7p22.1 microduplication syndrome and BD are dissimilar. However, one patient with 7p22.1 duplication had ASD as a phenotype.^41^ While BD and ASD are clinically distinct conditions, multiple lines of genetic evidence, including family studies, CNV analyses, and GWAS, suggest a partial overlap in their genetic risk factors.^42^ The 7p22.1 duplication observed in both disorders may exemplify how certain genetic variants may contribute to different, yet potentially related, psychiatric phenotypes.

*RNF216* encodes the E3 ubiquitin‐protein ligase that is expressed in a variety of human tissues at all stages of development.^43^ *RNF216* is a broadly expressed RBR E3 ligase, and loss‐of‐function mutations in *RNF216* are linked to neurodegenerative conditions such as Gordon–Holmes syndrome (GHS) and Huntington‐like disorders, characterized by hypogonadotropic hypogonadism, cerebellar ataxia, and dementia.^44^, ^45^, ^46^ GHS is an autosomal recessive adult‐onset neurodegenerative disorder characterized by progressive cognitive decline, dementia, and variable movement disorders such as ataxia and chorea. Since the Rnf216 protein is a regulator of synaptic strength,^47^ the dose‐dependent effect of *RNF216* might contribute to the neurological phenotype of duplicate CNV holders.

The identified CNV on 7p22.1 regions included genes other than *RNF216*, such as *RNF216‐IT1*, *MIR6874*, ENSG00000291099 (Lnc‐OCM‐3), *RN7SL556P*, and *OCM*. Therefore, the possibility that these genes contribute to the development of BD should be considered. *RNF216‐IT1* and *MIR6874* are encoded by an intron of *RNF216* and are noncoding RNA and microRNA, respectively. The functions of these genes are not well understood; however, the fact that they are encoded in an intron of *RNF216* suggests that they regulate the transcript levels of *RNF216*. *OCM* encodes the oncomodulin protein, also called β‐parvalbumin, which is a small EF‐hand Ca^2+^‐binding protein and belongs to the parvalbumin family.^48^ Parvalbumin is a calcium‐binding protein present in inhibitory interneurons that plays an essential role in regulating many physiological processes such as intracellular signaling and synaptic transmission.^49^ It is closely related to psychiatric disorders; however, its role as an oncomodulin is not well understood. However, OCM is not registered as a synapse‐related gene in the SynGO database.

In the gene‐set analysis, the integral component of the postsynaptic membrane (GO:0099055) and the postsynaptic membrane (GO:0045211) were significantly associated with BD. Other studies have reported that postsynapse‐related pathways are associated with BD.^50^ For example, Akula et al. performed a weighted gene coexpression network analysis using brain transcriptome data to search for gene networks that play an important role in the cause of BD.^51^ They identified BD‐associated modules enriched in the GO terms postsynaptic density (GO:0014069) and postsynaptic membrane (GO:0045211). BD has frequently been reported to be associated with postsynaptic density in other omics data^50^, ^51^, ^52^; this gene‐set analysis, however, did not confirm a significant association. Our results support the contribution of postsynaptic‐related pathways to the development of BD.

GO:0099055, confirmed to be associated with BD, included *GRM5*. The CNVs in *GRM5* were located at almost identical locations in the introns. To confirm whether this region functions as a regulatory region, we evaluated data from the PsychENCODE project.^53^ Among the CNV regions, H3K27ac peaks in the temporal cortex (ID: EH37E0232917) (Supplementary Fig. S1). Thus, deletion of a portion of the intron region by this CNV could result in the loss of the enhancer region in the temporal cortex and dysregulation of transcription. A study evaluating postmortem brains reported that expression of *GRM5* in Brodmann Area 9 was significantly reduced in patients with BD.^54^ These findings support a relationship between BD and *GRM5* expression.

In this study, CNVs were detected via an array‐based approach using aCGH. This approach has several limitations. First, short CNVs (<10 kb) were excluded from this study because they cannot be accurately detected by aCGH. The array‐based approach detects CNVs via probes; therefore, CNVs in regions of the human genome in which no probes are located were not included in this study. Second, detection of insertions is not possible using the array‐based approach. The sequencing‐based approach has a higher resolution than the array‐based approach and is expected to be able to detect CNVs at the single nucleotide level. Future large‐scale CNV studies using sequencing‐based approaches, such as WGS, are needed to elucidate the full picture of the contribution of rare CNVs to BD.

In conclusion, we successfully identified CNVs that overlapped with *RNF216*, a gene associated with BD. This CNV might be associated with other genetic disorders, such as 7p22.1 microduplication syndrome. We also observed a significant association with postsynaptic membranes. The findings of this study are expected to elucidate the pathophysiology of BD.

## Disclosure statement

Dr Kushima received research/grant support from AMED, MEXT/JSPS, Novartis Pharma, GlaxoSmithKline, Takeda Pharmaceutical Co., Ltd., the Hori Sciences and Arts Foundation, the Uehara Memorial Foundation, and the SENSHIN Medical Research Foundation. Dr Ozaki received research support and/or speakers' honoraria from or has served as a joint researcher with, or a consultant to, Sumitomo Pharma, Otsuka, KAITEKI, Takeda, Ricoh, Meiji Seika Pharma, Taisho Pharma, Mochida, Shionogi, Mitsubishi Tanabe, Tsumura, EA Pharma, Eli Lilly, Daiichi Sankyo, MSD, Lundbeck Japan, Viatris, Eisai, Mochida, Kyowa Pharmaceutical Industry, Nihon Medi‐Physics, TSUMURA, Kyowa Kirin, Janssen pharma, Yoshitomi Pharmaceutical, Nippon Chemiphar, Medical Review, Nippon Boehringer Ingelheim, Ono Pharmaceutical, Woolsey Pharmaceuticals, Nihon Mei‐Physics, and SUSMED, outside the submitted work. Dr Kato received grants or contracts from Sumitomo Pharma, Otsuka Pharmaceutical, Takeda Pharmaceutical, Eli Lilly Japan K.K., Teijin Pharma, Daiichi Sankyo, EA Pharma, and Eisai outside the submitted work. He also participated on a Data Safety Monitoring Board or Advisory Board of Glaxo‐SmithKline. Ryota Hashimoto, Norio Ozaki, and Masashi Ikeda are editorial board members of *Psychiatry and Clinical Neurosciences* and coauthors of this article. To minimize bias, they were excluded from all editorial decision‐making related to the acceptance of this article for publication. Tadafumi Kato is the Editor‐in‐Chief, Michio Suzuki is the Vice Editor‐in‐Chief, and Shigenomu Kanba is the Editor‐in‐Chief Emeritus of *Psychiatry and Clinical Neurosciences* and coauthors of this article. They were excluded from editorial decision‐making related to the acceptance and publication of this article. Editorial decision‐making was handled independently by Editor‐in‐Chief Hidehiko Takahashi to minimize bias. The other authors declare no biomedical financial interests or potential conflicts of interest.

## Author contributions

M.N., I.K., and N.O. designed the study. I.K. performed the aCGH. M.N., I.K., and Y.Y. analyzed the data. I.K., B.A., H.Ki., H.Ka., T.In., Y.T., N.T., M.Y., K.I., Y.N., S.I., N.I., T.Sa., K.N., T.Ok., R.H., H.Yam., Y.Yas., M.F., K.M., K.O., T.Sh., K.Ki., M.It., M.A., M.M., K.T., T.T., M.S., T.A., S.K., H.H., K.Ka., T.Ik., S.J., T.K., C.K., B.Y., S.N., Y.K., H.Yab., Y.O., S.T., F.U., T.Ob., Y.A., D.M., M.Ik., and N.O. recruited the participants and/or collected DNA samples or phenotype data. M.N. and I.K. wrote the first draft of the manuscript, and the other authors commented on and refined subsequent versions. The final manuscript was approved by all authors, who take full responsibility for its content.

## Supporting information


**Fig. S1.** Copy number variations (CNVs) detected in *GRM5* in patients with bipolar disorder (BD) and controls. UCSC Genome Browser view for the region chr11: 88,460,000‐89,070,000 bp. Genomic coordinates are based on hg38. The upper track shows the genomic locations of the detected duplications and deletions in the following order: patients with BD and healthy controls were measured using Agilent array comparative genome hybridization (aCGH), and validation samples were measured using NimblGen aCGH. Deletions are indicated by the red bars. Sample numbers do not correspond to the sample numbers in Figure 1. The lower track shows the gene annotations in GENCODE V43, DEL_CHR11_8173BAA3 of gnomAD SVs V4, and H3K27ac peaks for the temporal cortex (DER‐06) from the PsychENCODE project. Other variants registered in gnomAD are not shown.

**Table S1.** Sample characteristics.
**Table S2.** Results of the gene‐based analysis after addition of the presence or absence of pathogenic CNV as a covariate for *RNF216* and *GRM5*.
**Table S3.** Genes nominally associated with BD based on comprehensive gene‐based analysis.
**Table S4.** Frequencies of duplications in *RNF216* across different populations in gnomAD CNVs 4.0 (non‐neuro samples).
**Table S5.** Frequencies of CNV carriers in *RNF216* from the Japanese population in JCNVv1.
**Table S6.** Frequencies of deletions in *GRM5* across different populations in gnomAD SVs 4.0 (non‐neuro samples).

**Data S1.** Supplementary Methods.

## Data Availability

Data are available from the corresponding authors upon reasonable request.
