# Supplementary figures and images for: Copy number variations in RNF216 and postsynaptic membrane–associated genes are associated with bipolar disorder: a case‐control study in the Japanese population
Source: Psychiatry Clin Neurosci. 2024 Oct 15;79(1):12–20. doi: 10.1111/pcn.13752 (PMC11693978; doi:10.1111/pcn.13752)

## Slide 1
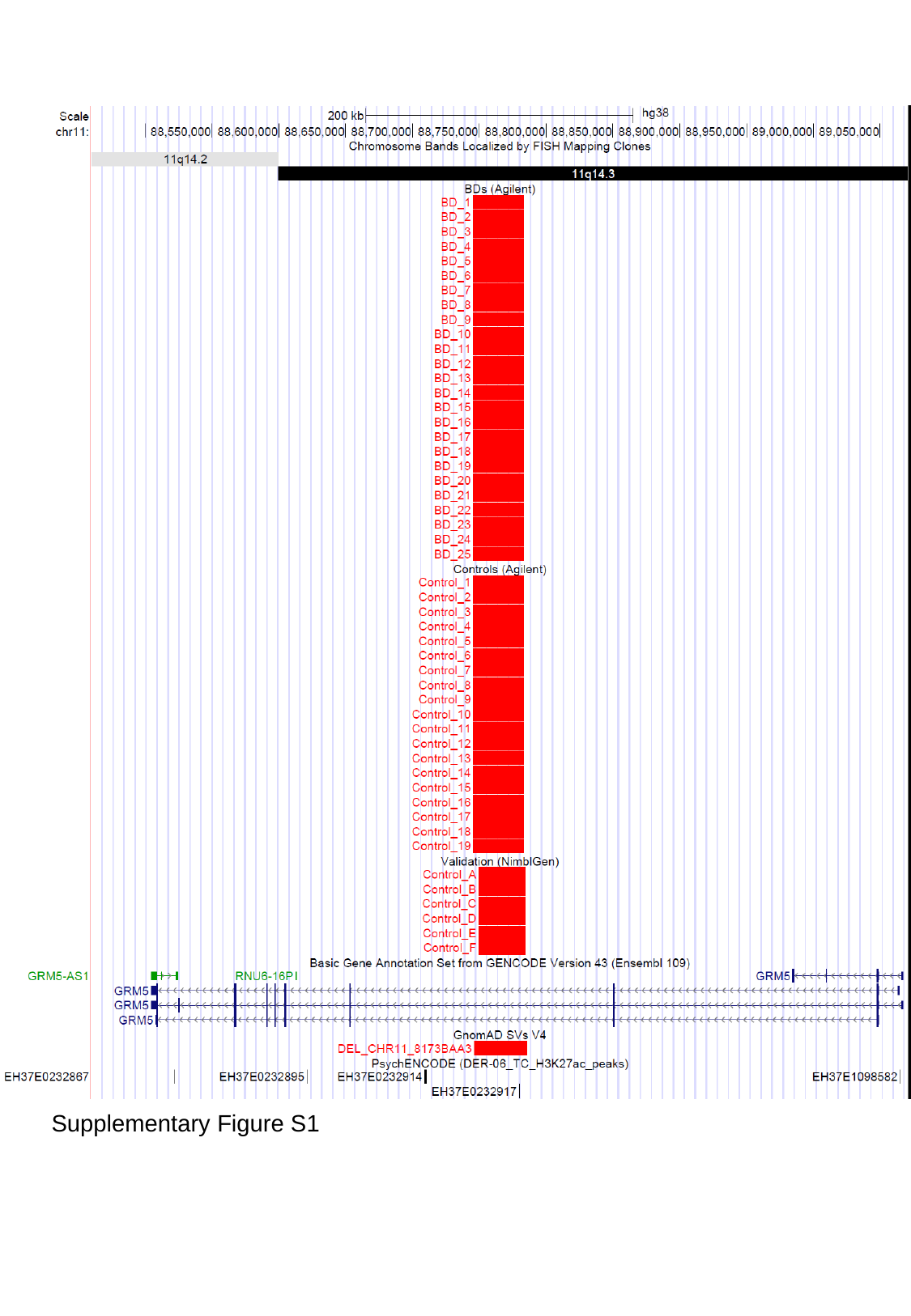

Supplementary Figure S1

Supplement: Supplementary file 1 — Fig. S1. Copy number variations (CNVs) detected in GRM5 in patients with bipolar disorder (BD) and controls. UCSC Genome Browser view for the region chr11: 88,460,000‐89,070,000 bp. Genomic coordinates are based on hg38. The upper track shows the genomic locations of the detected duplications and deletions in the following order: patients with BD and healthy controls were measured using Agilent array comparative genome hybridization (aCGH), and validation samples were measured using NimblGen aCGH. Deletions are indicated by the red bars. Sample numbers do not correspond to the sample numbers in Figure 1. The lower track shows the gene annotations in GENCODE V43, DEL_CHR11_8173BAA3 of gnomAD SVs V4, and H3K27ac peaks for the temporal cortex (DER‐06) from the PsychENCODE project. Other variants registered in gnomAD are not shown. [file PCN-79-12-s001.pptx]
