# Supplementary material for: Copy number variations in RNF216 and postsynaptic membrane–associated genes are associated with bipolar disorder: a case‐control study in the Japanese population
Source: Psychiatry Clin Neurosci. 2024 Oct 15;79(1):12–20. doi: 10.1111/pcn.13752 (PMC11693978; doi:10.1111/pcn.13752)
Supplement: Supplementary file 3 — Data S1. Supplementary Methods. [file PCN-79-12-s003.docx]

**Supplementary Methods**

***Quality control of aCGH and annotation of detected CNVs***

For quality control (QC), scores were calculated for each sample based on the statistical variance of the probe-to-probe log ratios. Lower QC scores indicated better quality results. Samples with a QC score >0.2, sex mismatch, or excessive autosomal CNV calls (subject to QC) were excluded. Next, we excluded CNV calls <10 kb; those with low probe density (<1 probe/30 kb), >70% overlap with segmental duplications (SDs), >10% overlap with CpG islands, or call *P*-values >1×10^-10^; and those on the Y chromosome. Common copy number variations (CNVs) (≧1% of our total samples) were filtered out. Large CNVs can be split using CNV-calling algorithms. To overcome this, adjacent CNV calls were merged using a custom script. Adjacent CNVs of the same type (i.e., deletion or duplication) were merged if they occurred in a single individual, and the gap was <50% of the entire length of the newly merged CNV. Furthermore, we filtered out common CNVs based on publicly available databases, such as Genome Aggregation Database (gnomAD) version 2.1 structural variation (SV)^1^ and SgD-CNV.^2^ The frequency of SV for non-neuro samples of gnomAD version 2.1 was obtained from https://gnomad.broadinstitute.org/downloads. The SgD-CNV data consisted of 6,533 individuals from three major Asian ethnic groups in Singapore: Chinese (n = 1, 917), Malays (n = 2, 399), and Indians (n = 2, 217).^2^ The CNV dataset was retrieved from the dbVar database (accession ID: nstd71). The frequency of the CNV carriers per nucleotide was calculated for each database. Genomic regions with a carrier frequency of ≧1% were identified as common CNV regions. In the CNV dataset of this study, CNVs that showed more than 30% overlap with common CNV regions were excluded. Finally, all statistical analyses were performed based on rare CNVs (< 1%). Each rare CNV was annotated using GENCODE release 43. CNVs in genes, regardless of exonic or intronic regions, were annotated as genic CNVs.

***Gene-based analysis adjusted for pathogenic CNVs***

We evaluated whether the identified genes were associated with bipolar disorder (BD) independently of known pathogenic CNVs. We identified pathogenic or likely pathogenic CNVs according to the guidelines of the American College of Medical Genetics (ACMG),^3, 4^ as in our previous study.^5^ We performed Firth’s bias-reduced logistic regression for two genes, *RNF216* and *GRM5*, and included sex and the presence or absence of pathogenic CNVs according to the ACMG criteria as covariates.

***Detection of CNVs Overlapping with RNF216 from the Whole-exome Sequencing (WES) Dataset of BD Trios***

To confirm the frequency of CNVs overlapping with *RNF216* in Japanese patients with BD, a WES dataset from Japanese BD trios was used.^6, 7^ WES was applied for target capture using SureSelect Human All Exon kits version 5 or 6 (Agilent Technologies) and was performed on DNA of children with BD and their parents. The dataset was obtained from National Bioscience Database Center Human Database, Japan, with the accession code JGAD000379. The authors attempted to detect CNVs overlapping with *RNF216* based on the WES dataset of BD trio. FASTQ files were processed using Trimmomatic (version 0.39), BWA-MEM (version 0.7.17),^8^ SAMtools,^9^ and GATK (version 4.5). CNV calls were made using GATK gCNV^10^ for the intersecting regions of SureSelect version 5 and 6.

***Gene-set Analysis***

To identify the synaptic biological pathways underlying BD pathogenesis, we tested the enrichment of rare genic CNVs in synaptic gene sets relative to that of all rare gene CNVs. To test the enrichment in each synaptic gene set, we used Firth’s logistic regression analysis. The dependent variable was the case versus control status. The independent variables were the number of genes within a given gene set that overlapped with rare genic CNVs, and sex, total length of rare CNVs, and number of rare CNVs were used as covariates. This method is robust against case-control differences in the total length of rare CNVs, number of rare CNVs, and systematic differences in gene size.^11^ Enrichment in cases was shown as one-sided empirical *P*-values using 10,000 permutations and swapping the case-control status. *P*-values were corrected for false discovery rate (FDR) based on the Benjamini and Hochberg method^12^ to correct for multiple testing. A gene set was considered significant when the *Q*-value was <0.10. Synaptic gene sets from SynGO version 1.2 were used in this study. We analyzed 33 SynGO gene sets with at least 20 overlapping genes in our rare CNV dataset.

**References**

1. Collins RL, Brand H, Karczewski KJ et al. A structural variation reference for medical and population genetics. *Nature* 2020; **581:** 444-451.

2. Xu H, Poh WT, Sim X et al. SgD-CNV, a database for common and rare copy number variants in three Asian populations. *Hum Mutat* 2011; **32:** 1341-9.

3. Brandt T, Sack LM, Arjona D et al. Adapting ACMG/AMP sequence variant classification guidelines for single-gene copy number variants. *Genet Med* 2020; **22:** 336-344.

4. Riggs ER, Andersen EF, Cherry AM et al. Technical standards for the interpretation and reporting of constitutional copy-number variants: a joint consensus recommendation of the American College of Medical Genetics and Genomics (ACMG) and the Clinical Genome Resource (ClinGen). *Genet Med* 2020; **22:** 245-257.

5. Kushima I, Nakatochi M, Aleksic B et al. Cross-Disorder Analysis of Genic and Regulatory Copy Number Variations in Bipolar Disorder, Schizophrenia, and Autism Spectrum Disorder. *Biol Psychiatry* 2022; **92:** 362-374.

6. Kataoka M, Matoba N, Sawada T et al. Exome sequencing for bipolar disorder points to roles of de novo loss-of-function and protein-altering mutations. *Mol Psychiatry* 2016; **21:** 885-893.

7. Nishioka M, Kazuno AA, Nakamura T et al. Systematic analysis of exonic germline and postzygotic de novo mutations in bipolar disorder. *Nat Commun* 2021; **12:** 3750.

8. Li H, Durbin R. Fast and accurate short read alignment with Burrows-Wheeler transform. *Bioinformatics* 2009; **25:** 1754-1760.

9. Li H, Handsaker B, Wysoker A et al. The sequence alignment/map format and SAMtools. *Bioinformatics* 2009; **25:** 2078-2079.

10. Babadi M, Fu JM, Lee SK et al. GATK-gCNV enables the discovery of rare copy number variants from exome sequencing data. *Nat Genet* 2023; **55:** 1589-1597.

11. Raychaudhuri S, Korn JM, McCarroll SA et al. Accurately assessing the risk of schizophrenia conferred by rare copy-number variation affecting genes with brain function. *PLoS Genet* 2010; **6:** e1001097.

12. Benjamini Y, Hochberg Y. Controlling the false discovery rate - a practical and powerful approach to multiple testing. *J Roy Stat Soc B-Methodol* 1995; **57:** 289-300.
